# Inhabited or Uninhabited? Pitfalls in the Interpretation of Possible Chemical Signatures of Extraterrestrial Life

**DOI:** 10.3389/fmicb.2017.01622

**Published:** 2017-08-25

**Authors:** Stefan Fox, Henry Strasdeit

**Affiliations:** Department of Bioinorganic Chemistry, Institute of Chemistry, University of Hohenheim Stuttgart, Germany

**Keywords:** false positives, false negatives, contaminants, abiotic organics, biomolecules, inorganic metabolites, Mars, icy moons

## Abstract

The “Rare Earth” hypothesis—put forward by Ward and Brownlee in their 2000 book of the same title—states that prokaryote-type organisms may be common in the universe but animals and higher plants are exceedingly rare. If this idea is correct, the search for extraterrestrial life is essentially the search for microorganisms. Various indicators may be used to detect extant or extinct microbial life beyond Earth. Among them are chemical biosignatures, such as biomolecules and stable isotope ratios. The present minireview focuses on the major problems associated with the identification of chemical biosignatures. Two main types of misinterpretation are distinguished, namely false positive and false negative results. The former can be caused by terrestrial biogenic contaminants or by abiotic products. Terrestrial contamination is a common problem in space missions that search for biosignatures on other planets and moons. Abiotic organics can lead to false positive results if erroneously interpreted as biomolecules, but also to false negatives, for example when an abiotic source obscures a less productive biological one. In principle, all types of putative chemical biosignatures are prone to misinterpretation. Some, however, are more reliable (“stronger”) than others. These include: (i) homochiral polymers of defined length and sequence, comparable to proteins and polynucleotides; (ii) enantiopure compounds; (iii) the existence of only a subset of molecules when abiotic syntheses would produce a continuous range of molecules; the proteinogenic amino acids constitute such a subset. These considerations are particularly important for life detection missions to solar system bodies such as Mars, Europa, and Enceladus.

## Introduction

Life can leave different kinds of signatures. Fossil evidence of microorganisms, for example, can be morphological (e.g., fossilized cells) or chemical (organic, mineralogical, elemental, isotopic; Westall and Cavalazzi, 2011). Biosignatures by definition are of biological origin. However, even the discrimination between fossilized cells and abiogenic structures, which might be expected to be straightforward, can require special methods and sophisticated instrumentation (Westall et al., 2011). A cautionary example is the erroneous interpretation of filamentous structures in 3.46 Ga old rocks from the Pilbara Craton of northwestern Australia. These structures were originally believed to be cyanobacterial microfossils (Schopf, 1993). This view was later challenged (“Schopf–Brasier controversy”; see Hazen, 2005). Strong evidence against the biogenicity of these structures comes from chemistry. Chemical analysis by Raman spectroscopy revealed that morphologically similar structures from the same locality as the original specimens were in fact quartz and haematite-filled fractures (Marshall et al., 2011; Marshall and Marshall, 2013). Interestingly, carbonaceous material, though not associated with these structures, occurs disseminated in the surrounding mineral matrix. It could be a degradation product of biomolecules, in other words, a chemical biosignature.

Searching for chemical constituents of organisms and other chemical fingerprints of life is not only important in studies of ancient traces of life on Earth but is also a promising approach to establish whether other planets and moons are, or were, inhabited. Currently, the Sample Analysis at Mars (SAM) suite of instruments on NASA’s Curiosity rover performs chemical analyses on Mars to assess the planet’s habitability and to possibly find evidence of extant or extinct life (Mahaffy et al., 2012). In the near future, ESA’s ExoMars rover and NASA’s Mars 2020 rover will join the search for chemical biosignatures on the red planet (Beegle et al., 2015; Goetz et al., 2016). Chemical analyses of the (sub)surfaces and plumes of icy moons, such as Enceladus and Europa, could answer the question of whether these moons’ subsurface oceans harbor life. Several studies on missions to Enceladus and Europa have been proposed. Many include the search for chemical biosignatures—for example, “Enceladus Life Finder” (ELF; Lunine et al., 2015), “Testing the Habitability of Enceladus’s Ocean” (THEO; MacKenzie et al., 2016), “Enceladus Explorer” (EnEx; Konstantinidis et al., 2015), and “Europa Lander” (Hand et al., 2017). The realization of such missions, however, is still some way off.

Among the advantages of chemical biosignatures is that often well-established analytical methods such as Raman spectroscopy and gas chromatography/mass spectrometry can be used for detection and identification (Rodier et al., 2005; Villar and Edwards, 2006; Poinot and Geffroy-Rodier, 2015). Furthermore, certain “biomarkers”—a term that in a narrow sense refers to biomolecules or specific degradation products thereof—can persist over long periods of time. Examples of such “molecular fossils” are 1.64 Ga old carotenoid derivatives (Lee and Brocks, 2011) and degradation products of chlorophylls and hemes (geoporphyrins; Callot and Ocampo, 2000) which have been reported, for example, from ∼500 Ma old oil shales (Serebrennikova and Mozzhelina, 1994). Hence, the extraordinary stability of certain molecular fossils opens the prospect of detecting chemical traces of life on other planets and moons even if it became extinct a long time ago. Accurate dating, however, can be difficult because the molecules need not necessarily be of the same age as the surrounding geological matrix. A good example is 2-methylhopanes and steranes from 2.7 Ga old shales from Australia. These molecules, which derive from membrane lipids, are regarded as characteristic biomarkers for bacteria and eukaryotes, respectively. Therefore, it was originally concluded that eukaryotes and possibly oxygen-producing cyanobacteria already existed 2.7 Ga ago (Brocks et al., 1999). This conclusion was refuted by Rasmussen et al. (2008) who demonstrated that the biomarkers were not indigenous but entered the rock at least ∼0.5 Ga later.

It is often difficult to identify chemical biosignatures unambiguously because they are prone to various types of misinterpretation. In this context, two main categories can be distinguished, namely (i) chemical compounds and properties that are mistakenly considered as products of extraterrestrial life (“false positives”) and (ii) chemical biosignatures of extraterrestrial origin that are not recognized as such (“false negatives”). The category of false positive biosignatures may be further subdivided into terrestrial biogenic contaminants and products of non-biological processes. This minireview gives examples of potential pitfalls in the search for extraterrestrial chemical biosignatures. It is not meant to be comprehensive. Accordingly, some aspects of the topic are not addressed—for example, unusual concentrations of elements that may or may not result from biological processing.

## False Positives I: Terrestrial Contaminants

Terrestrial biological contaminants could be unambiguously identified in some meteorites. For example, Brinton et al. (1998) found that all five samples of Antarctic micrometeorites they studied contained terrestrial amino acids. Similarly, in some Apollo lunar regolith samples the amino acids alanine, aspartic acid, glutamic acid, serine, threonine, and valine showed a clear L-enantiomeric excess, implying at least a contribution from terrestrial biology (Elsila et al., 2016). The contaminants were present despite the fact that the samples had been stored under NASA curation since their collection. Such terrestrial biological contamination of samples from space bears the risk of false positive detection of extraterrestrial molecules (or even biosignatures). However, it is necessary to ascertain that the suspicious molecules are really of terrestrial origin. In the case of meteorites, this has been accomplished, for example, by comparing the meteoritic amino acid composition with that of the environment from which the meteorite was collected (Brinton et al., 1998; Kminek et al., 2002).

In a certain sense, the contamination of extraterrestrial objects on Earth is related to the contamination of other planets and moons by spacecrafts. The latter process, called “forward contamination,” is an important aspect of planetary protection (Race, 1995; Rummel, 2001) and a persistent problem in space missions (Debus, 2006). Although the main goal is to protect extraterrestrial environments against viable organisms from Earth, terrestrial extracellular biomolecules may also be deleterious, at least to the scientific exploration of these environments. Adenosine triphosphate (ATP), for example, may stay intact for 100s of sols in martian UV climate under certain conditions such as shielding by the spacecraft or in global dust storms (Schuerger et al., 2008). Thus, ATP and possibly other terrestrial biomolecules could remain detectable on the surface of a spacecraft throughout the entire duration of a mission. Again, here is a possible source of false positives.

In addition to biological contaminants, man-made substances may also be critical. These include chemicals used in production and cleaning processes and in onboard analyses (e.g., reagents for GC–MS). They can be difficult to discern from indigenous organics or products thereof. For example, the source of the chlorinated methane derivatives CH_x_Cl_y_ that have been detected in the martian soil by the Viking Landers and more recently by the Mars Science Laboratory (MSL) rover Curiosity is unclear. They may originate from anthropogenic sources, meteoritic organics in the regolith or indigenous martian molecules (Leshin et al., 2013; Ming et al., 2014). There is a comprehensive paper by Summons et al. (2014) that deals with possible organic contaminations of martian samples with special regard to NASA’s Mars 2020 rover mission, which is planned to cache samples for a future return to Earth.

## False Positives II: Abiotic Organics

Abiotically formed organic molecules are another potential source of false positive biosignatures. A huge number of them have been found in carbonaceous meteorites (Cronin and Chang, 1993; Schmitt-Kopplin et al., 2010), in the interstellar medium (Irvine, 1998; Kwok, 2012) and in laboratory simulations of early Earth chemistry (Miller, 1993, 1998). This diversity shows that natural abiotic pathways exist to “simple” organic compounds from virtually all classes. Porphyrins, for example, were originally regarded as “ideal biomarkers” (Suo et al., 2007), partly because no experimental evidence for their abiotic formation existed. In recent years, however, abiotic routes to these molecules have been discovered, so that we now need to look more critically at the role of porphyrins as biosignatures (Fox and Strasdeit, 2013).

There are different approaches that can help to distinguish biotic from abiotic molecules. One is to look at the relative abundances of monomers of the same compound class, such as amino acids [“monomer abundance distribution biosignature” (MADB); Dorn et al., 2011]. This approach is based on the idea that “abiotic synthesis forms the compounds that are *easy to make*. Organisms, on the other hand, make the compounds *that they need* in order to survive and compete” (Dorn, 2005). If this holds universally, which seems to be a reasonable assumption, then the differences in the distribution patterns would be a powerful tool to identify extraterrestrial chemical biosignatures.

Another approach uses the fact that abiotic synthesis yields a continuous range of members of a compound class, while organisms synthesize only a subset of the possible molecules (“Lego Principle”; McKay, 2004). The α-amino acids of the type (^+^H_3_N)CHR(CO_2_^-^) are a good example. In Miller-Urey type spark discharge experiments, the members with R = Me, Et, *n*-Pr, and *iso*-Pr form together (Johnson et al., 2008). In addition to these four, the amino acids with R = *n*-Bu, *iso*-Bu, *sec*-Bu, *tert*-Bu, and the eight pentyl isomers were detected in the Murchison meteorite (Meierhenrich, 2008). However, only four out of these 16 homologs occur in proteins, namely alanine (R = Me), valine (R = *iso*-Pr), leucine (R = *iso*-Bu), and isoleucine (R = *sec*-Bu). In the Lego Principle, the mere fact that a discontinuous instead of a continuous distribution pattern exists is indicative of a biological origin. Details of the subset play a subordinate role (Davies et al., 2009). This is advantageous because an extraterrestrial organism may use subsets different from those of terrestrial organisms. Nevertheless, knowing the exact nature of the subset may be important to exclude terrestrial biological contamination. Biological homochirality also fits into the Lego Principle (see below).

In addition to the chemical structures, stable isotope ratios have been used to infer the source of organic compounds. A strong depletion in ^13^C, for example, is often interpreted as an indication of biogenic origin. However, results of laboratory experiments suggest that abiotic Fischer–Tropsch-type syntheses in hydrothermal settings can produce degrees of ^13^C depletion similar to those found in organisms (McCollom and Seewald, 2006). Therefore, the carbon isotope ratio alone may not be particularly suitable to identify biological carbon and may lead to false positive identification of biomolecules in extraterrestrial samples. An actual case is the methane that has been detected in trace concentrations in the martian atmosphere. The release of this gas from carbonaceous meteorites by ultraviolet radiation—a possible source on Mars—has been studied experimentally by Keppler et al. (2012). The authors found that the deuterium isotopic signature clearly confirmed the extraterrestrial origin of methane released from the Murchison meteorite. In contrast, the δ^13^C values were in the range of the carbon isotopic signatures of methane from terrestrial biogenic sources. Some of the δ^13^C data from NOMAD might be similarly misleading. NOMAD is part of the ExoMars Trace Gas Orbiter payload and designed to spectroscopically determine isotope ratios in atmospheric molecules such as methane and carbon dioxide (Vandaele et al., 2015). Fortunately, the combined measurement of different isotopologs (for example, ^13^CH_4_, CH_3_D and ^13^CO_2_, ^17^OCO, ^18^OCO, C^18^O_2_) is planned. This will certainly reduce the danger of misinterpretation of results.

## False Negatives

Situations are conceivable where a substantial risk of false negative chemical biosignatures exists. For example, when a molecule simultaneously has a biotic and an abiotic source and the biotic production rate is low, the partially biological origin of this molecule may be overlooked. This situation might occur for methane on other planets and moons. In fact, on Earth, methane is not only produced by archaea and by decomposition of organic matter but also by magmatic processes and gas–water–rock interactions (Horita and Berndt, 1999; Etiope and Sherwood Lollar, 2013). Terrestrial hydrothermal systems are typical environments where both biological and non-biological methane sources coexist (Bradley, 2016). In this context, it is interesting to note that hydrothermal activity probably also occurs on the ocean floor of Saturn’s moon Enceladus (Hsu et al., 2015). Other compounds that have biotic and abiotic sources are the oxalate minerals, such as whewellite (CaC_2_O_4_⋅H_2_O). Oxalates on Mars, for example, may originate from meteoritic organic material as well as from extant or extinct martian life (Applin et al., 2015).

Some structural criteria that are useful in identifying molecules of terrestrial biological origin may fail when applied to extraterrestrial samples. For example, the odd-over-even predominance found in many sedimentary *n*-alkanes indicates an origin from biomolecules, namely fatty acids with even numbers of C atoms. It is routinely regarded as diagnostic of a biological origin (for example, of a 3.5 Ga old kerogen; Derenne et al., 2008). Rare cases of even-over-odd predominance in *n*-alkanes have been reported (Nishimura and Baker, 1986). Nevertheless, according to the Lego Principle (see above), the mere existence of a predominance is a strong biosignature—at least in terrestrial samples. Fatty acids of an extraterrestrial organism, however, may be neither preferentially even- nor odd-chain. Thus, their biological origin can remain unrecognized if the even–odd pattern is the only criterion used. This false negative may possibly be avoided by searching for structural features that can occur in biological fatty acids, such as unsaturation, chain branching, and others (Georgiou and Deamer, 2014). In general, an alien biochemistry, even if carbon-based, could be very different from the one we know (Schulze-Makuch and Irwin, 2008). Therefore, its molecules may not be readily recognized as biosignatures.

Extensive destruction of biomolecules by heat and/or pressure (for example, in diagenesis; Hatcher and Clifford, 1997; Killops and Killops, 2005; Wakeham and Canuel, 2006; Vandenbroucke and Largeau, 2007; Arndt et al., 2013) or by energetic radiation (Ward, 1985; Garrison, 1987; Kempner, 1993) can lead to inconclusive or even false negative results because the decomposition products may not reveal their origin. Unfavorable properties could make some decomposition products difficult to detect. The glycine thermomelanoid may be a good example. Its composition is not well-defined and depends on the formation temperature, the material has a low solubility, and its spectra are not particularly informative (Fox et al., 2015).

Recently, a promising technique for the detection of viable microorganisms has been described (Kasas et al., 2015). It is based on the nanoscale fluctuations that a living cell shows due to its metabolic activity. This method is independent of specific biomolecules because it observes a physical manifestation of the *overall* metabolism. Therefore, it avoids the problem of false negatives that can occur with identifying extraterrestrial biomolecules (see above). Of course, this technique can only detect sufficiently metabolically active cells, but not dead or dormant ones.

Finally, it is important to emphasize that negative results in the detection of a chemical biosignature should not be equated with its absence. The biosignature may be present but in concentrations below the analytical detection limit, or the respective environment may prevent its detection. Here, the sentence “Absence of evidence is not evidence of absence” applies (Sagan, 1995).

## Biopolymers

It is highly unlikely that a natural abiotic process generates long chain molecules that have precisely defined lengths, ordered sequences, and homochiral building blocks. Therefore, proteins and nucleic acids can certainly be regarded as strong chemical biosignatures. Alien life forms, too, will probably be built on ordered polymers, but not necessarily on polypeptides and polynucleotides (Schulze-Makuch and Irwin, 2008).

Biopolymers break down more or less rapidly outside of living organisms. The decomposition often proceeds until the ultimately biological origin is no longer identifiable (false negatives, see above). In exceptional cases, however, larger fragments can still be intact after very long periods of time. With respect to long-term stability, proteins are more promising than DNA (Cappellini et al., 2014). An intriguing example of very old protein fragments is collagen peptides that have been detected in bone matrix from a ∼80 Ma old *Brachylophosaurus canadensis* specimen (Schroeter et al., 2017). Hence, it is perhaps not over-optimistic to assume that biopolymer fragments can have long lifetimes on other planetary bodies, too, if the conditions are favorable. However, even on Earth unambiguous identification is difficult, as shown by a recent example (Arnaud, 2017; Lee et al., 2017).

## Homochiralty And Enantiopurity

Homochiral and enantiopure molecules each form “subsets” in terms of the Lego Principle (see above). Homochirality and, in the case of individual compounds, enantiopurity represent powerful chemical biosignatures. No natural non-biological processes that generate them have been observed in nature, but there are some indications that, at least in rare instances, natural abiotic compounds might be enantiopure. For example, there is a single case where, under laboratory conditions, a small enantiomeric excess of an amino acid was amplified to near enantiopurity (>99%; Klussmann et al., 2006). This amino acid was serine under solid–liquid equilibrium conditions in water at the eutectic point. However, for all other amino acids tested, enantiopurity was not achieved. Also, this mechanism will not work with chiral compounds that crystallize as conglomerates (i.e., mixtures of pure L and pure D crystals). Because of the special conditions and compounds necessary, it is unclear if this physical process is relevant to the generation of enantiopurity (i.e., enantiomeric excesses near 100%) in extraterrestrial environments.

In addition, as-yet-undiscovered abiotic chemical reactions that yield enantiopure products might exist in nature. In fact, a laboratory reaction is known that can amplify enantiomeric excesses as low as 10^-5^ % to virtual enantiopurity (>99.5%; Soai and Kawasaki, 2008). This so-called Soai reaction is, however, highly water-sensitive and therefore not relevant to habitable environments.

Low to moderate enantiomeric excesses, as they occur, for example, in meteoritic α,α-dialkyl amino acids (Pizzarello and Cronin, 2000), are definitely not indicative of a biological origin. On the other hand, a lack of enantiopurity can be a false-negative result because the initial enantiopurity could have been lost by racemization, a process well-known for the proteinogenic L-amino acids (Bada and Schroeder, 1975; Bada, 1985). Furthermore, one should not discard the possibility that an extraterrestrial organism synthesizes both enantiomers. In fact, terrestrial bacteria produce diverse D-amino acids (e.g., D-Ala, D-Glu, D-Leu, D-Met, D-Phe, and D-Tyr) which have effects on the peptidoglycan of the cell wall, both directly by incorporation into the polymer and indirectly by regulating enzymes that synthesize and modify peptidoglycan (Höltje, 1998; Lam et al., 2009). Another intriguing example from terrestrial life is the simultaneous presence of L- and D-isovaline in some fungal peptides (Degenkolb et al., 2007).

## Inorganic Metabolic Products

Certain inorganic compounds—mainly biominerals and some simple gasses—can also serve as indicators of biological activity. They may, however, face the same problem of ambiguity as organic biosignatures. A well-known example is the magnetite crystals in the martian meteorite ALH84001. They have been interpreted as a biomineral similar to the magnetosome crystals of terrestrial magnetotactic bacteria (McKay et al., 1996). Although this interpretation cannot be excluded, it remains inconclusive (Weiss et al., 2004). Other features of ALH84001 which were originally taken as evidence for former life on Mars appear to be even less conclusive (Jakosky et al., 2007). These include (i) ovoid structures that were interpreted as microbial fossils but appear to be too small for bacterial cells, do not meet the criteria for microbial fossils (Westall and Cavalazzi, 2011) and can be explained as products of aqueous corrosion of carbonates; and (ii) organic compounds that were regarded as chemical biosignatures but were mainly of terrestrial origin, and those of martian origin could have been formed by non-biological processes.

Chemical disequilibria that would rapidly disappear if the input from metabolic activity ceases can be relatively robust biosignatures. For example, considering the O_2_ concentration, the methane concentration in the terrestrial atmosphere appears to be several orders of magnitude too high (Sagan, 1970). A high O_2_ concentration, if discovered in the atmosphere of a rocky exoplanet with moderate surface temperature, is often regarded as a good indicator of biological photosynthesis (Léger et al., 2011). This view, however, has been challenged by the argument that abiotic water photolysis on a habitable-zone planet can also produce an oxygen-dominated atmosphere and thus may result in a false positive for life (Wordsworth and Pierrehumbert, 2014). According to Stüeken et al. (2016), it is unlikely that high levels of *abiotic* O_2_ can coexist with high levels of N_2_. In other words, the simultaneous presence of large amounts of both O_2_ and N_2_ indicates an aerobic biosphere and reduces the risk of false positives.

## Concluding Remarks

In summary, it can be said that no putative chemical biosignature is inherently safe from misinterpretation. In the coming years, life-detection missions will focus on Mars and the icy moons of Jupiter and Saturn. Spacecrafts going to these destinations should not only be capable of performing sophisticated chemical analyses but also provide as much as possible contextual information, which is essential to identify chemical biosignatures. Returning extraterrestrial samples to Earth for analysis would be a major step forward. Indeed, sample return from the surface of Mars or the plumes of Enceladus is now within the realm of the technologically feasible.

## Author Contributions

SF and HS conceived, wrote and edited the minireview, and approved it for publication.

## Conflict of Interest Statement

The authors declare that the research was conducted in the absence of any commercial or financial relationships that could be construed as a potential conflict of interest.
